# CYP1A1 Ile462Val polymorphism and colorectal cancer risk in Polish patients

**DOI:** 10.1007/s12032-014-0072-y

**Published:** 2014-06-18

**Authors:** Justyna Gil, Paweł Gaj, Błażej Misiak, Jerzy Ostrowski, Pawel Karpinski, Alicja Jarczyńska, Wojciech Kielan, Maria Malgorzata Sasiadek

**Affiliations:** 1 Department of Genetics, Wroclaw Medical University, Marcinkowskiego 1, 50-368 Wroclaw, Poland; 2 Department of Gastroenterology and Hepatology, Medical Center for Postgraduate Education, Warsaw, Poland; 3 Department of Immunology, Center of Biostructure Research, Medical University of Warsaw, Warsaw, Poland; 4 2nd Department of General and Oncological Surgery, Wroclaw Medical University, Wroclaw, Poland

**Keywords:** Colorectal cancer, Xenobiotic-metabolizing enzymes, DNA repair, Single nucleotide polymorphisms SNPs

## Abstract

Colorectal cancer (CRC) is an epidemiological problem of a great importance in Poland; each year approximately 14,600 new cases of the disease are diagnosed. Mortality associated with CRC reaches approximately 10,400 cases per year (according to the National Cancer Registry). The 5-year survival rate is approximately 25 %, which is one of the lowest rates in Europe. The etiology of sporadic colorectal cancer (CRC) is multifactorial and has been attributed to an interplay between both environmental and genetic risk factors. In addition, there is a general consensus that genetic factors may modulate the influence of environmental insults. Following these assumptions, we performed a study on widely described polymorphisms in xenobiotic-metabolizing enzymes and DNA repair genes which may influence individual susceptibility to cancer. We selected five candidate polymorphisms in following genes: *ERCC1* Asp118Asn (rs11615), *XPC* i11C/A (rs2279017), *XRCC3* Met241Thr (rs861539) CYP1A1 Ile462Val (rs1048943) and *NAT2* A803G (rs1208) and assessed the importance of chosen SNPs on groups consisting of 478 CRC patients and 404 controls. Only CYP1A1 Ile462Val was statistically significant in CRC patients over 50 years old: OR 2.05 (1.29–3.28); *p* = 1.25E−02 and this association was more pronounced in the female group of CRC patients after the age of 50: OR 2.72 (1.43–5.14); *p* = 1.14E−02.

**Electronic supplementary material:**

The online version of this article (doi:10.1007/s12032-014-0072-y) contains supplementary material, which is available to authorized users.

## Introduction

Every year in developed countries, including Poland, a growing number of cases of sporadic colorectal cancer is observed. This tumor is still associated with a high mortality rate in patients. Several lines of evidence indicate that lifestyle factors including cigarette smoking, alcohol consumption and dietary habits may contribute to sporadic colorectal cancer (CRC) risk [1]. In addition, genetic variations in different biological pathways may modulate the influence of environmental insults in a gene × environment (G × E) manner [2].

Up-to-date, a plethora of low-penetrance single nucleotide polymorphisms (SNPs) has been linked to the molecular background of CRC [3]. Numerous studies have been dedicated to the role of genetic variants in genetic/biochemical pathways such as xenobiotic-metabolizing enzymes or DNA repair networks, which may modify influence of personal vulnerability to environmental exposures and consequently individual susceptibility to CRC [4–7].

Xenobiotic-metabolizing enzymes (XMEs) create a first line of protection against numerous mutagenic agents [8]. An activation of chemical pro-carcinogens is mediated mainly by cytochrome P-450 enzymes (phase I biometabolism of xenobiotics), while an elimination of environmental chemicals proceeds through the complex detoxification mechanisms and is associated with phase II biometabolic enzymes [9]. Among phase I enzymes, CYP1A1 polymorphisms have been extensively studied in the etiology of different cancers such as breast, prostate, lung [9–11]. In turn, the impact of genetic variation in phase II xenobiotic clearance enzymes has been attributed among others to such genes as *NAT2* (*N*-acetyltransferase 2) and *GSTT1* (glutathione S-transferase T1) [12].

The DNA repair network is a key protection against DNA-damaging carcinogenesis. A DNA repair system consists of a multitude of genes acting on several pathways specialized in repair of distinct types of DNA damage. The base excision repair (BER) pathway is implicated in counteracting the adversity of reactive oxygen species and ensured by the 8-oxoguanine DNA glycosylase (*OGG1*) gene and *XRCC1* gene [13]. The nucleotide excision repair (NER), involved in scavenging ultraviolet and xenobiotic-induced DNA modifications, comprises an activity of xeroderma pigmentosum (*XP*) genes and the excision repair cross-complementing group genes (*ERCC*) [14]. Finally, homologous recombination repair (HR) is responsible for repairing double-strand DNA damages and is linked, among others, to activity of nibrin 1 (*NBS1*) and *Rad51* [15].

Results of numerous studies on the association among genes involved in aforementioned pathways were often contradictory with insufficient statistical power. Moreover, a separate assessment of various genes did not allow to ensure the comprehensive insight into the molecular etiology of cancer including CRC.

In the present study, we performed an analysis on a Polish cohort consisting of 478 patients and 404 controls. Participants of the study originate from southwest (Lower Silesia) and central Poland. In our study, we analyzed two polymorphisms in XMEs: CYP1A1 (rs1048943) and *NAT2* A803G (rs1208) and three in DNA repair genes *ERCC1* Asp118Asn (rs11615)*, XRCC3* Met241Thr (rs861539)*, XPC* i11C/A (rs2279017). The selection of mentioned polymorphisms was supported by analysis of avaliable/most current literature data [16–28] as well as our preliminary studies concerning *CYP1A1* and *NAT2* polymorphisms (unpulished).

## Materials and methods

### Subjects

#### Colorectal cancer patients’ group

In total, 478 CRC patients were analyzed. Blood samples from 110 sporadic CRC patients were obtained from 2nd Department of General and Oncological Surgery and 1st Department of General Gastroenterological and Endocrine Surgery, Wroclaw Medical University, Lower Silesia (65,64 ± 10,10) (Supplementary Fig. 1, Supplementary Table 1). DNA from latter 368 patients with sporadic CRC was obtained from Warsaw Centre of Oncology-Institute, central Poland (aged 48.85 ± 12.23) (Supplementary Table 2 and Supplementary Fig. 2).

#### Control group

In total 404 controls were analyzed. Hundered healthy controls were enrolled at Department of Internal Diseases, Clinical Hospital, Swiebodzice, Lower Silesia as described previously [16]. This group is briefly presented in Supplementary Table 1 and Supplementary Fig. 1. Further 304 healthy volunteers (from central Poland) were tested negative (screening colonoscopy) for any abnormalities in the lower gastrointestinal tract. Familial and individual history regarding neoplasia was negative within the group of controls. All participants were Caucasians; all completed and signed an informed consent. They were aged 58.35 ± 4.92 (Supplementary Table 2 and Supplementary Fig. 2). The study was approved by the local Ethics Committee.

#### Combined groups, demographic data

Five selected polymorphisms were analyzed on a combined group consisting of 478 CRC cases versus 404 controls. The mean age of CRC patients was 52.75 ± 13.73 and controls 62.45 ± 9.15 (Supplementary Table 3 and Supplementary Fig. 3).

### Analysis of studied polymorphisms

Genomic DNA from Lower Silesian participants was obtained from peripheral blood lymphocytes using standard phenol–chloroform extraction and ethanol precipitation. Genotyping of studied polymorphisms was performed using polymerase chain reaction and restriction fragment length polymorphism (PCR–RFLP) method described elsewhere [8, 16].

The DNA for the Centre of Oncology-Institute (Warsaw) group was extracted using the QIAamp DNA Blood Mini Kit (QIAGEN). Genotyping of selected polymorphisms was done using the TaqMan^®^ SNP Genotyping Assays (Life Technologies), SensiMix II Probe Kit (Bioline) and the ABI Prism^®^ 7900HT real-time PCR system.

### Statistics

Before running the association analysis, the PLINK v1.07 software was used to rule out any deviations from the Hardy–Weinberg equilibrium in the control groups of individuals.

To asses statistical power of the analysis for each cohort, *PS: Power and Sample Size Calculation version 3.0* software was used taking into account common and rare variants [29].

The selected polymorphisms were analyzed using both the Fisher’s exact test using the PLINK software, and the logistic regression analysis was performed in *R*-*project* accounting for the additive model of gene action. The logistic regression model included patients’ gender as an additional independent variable. The reference (index) level for the sex variable was set to *female*.

To evaluate the cumulative performance of the studied polymorphic variants to discriminate between high and low CRC risk individuals, the ROC (receiver operating characteristic) curves were plotted and the AUC (area under the curve) parameters were computed using the *epicalc* package of the *R*-*project* [30].

## Results

For statistical analysis, we took into account the aforementioned Wroclaw Medical University (WMU) cohort, an independent group of CRC cases and controls collected at Warsaw Center of Oncology-Institute (COI) as well as both of the groups combined together. The respective sub-studies reached various levels of statistical power to identify associations for alleles found in the general population at high (p0 = 0.5) and low (p0 = 0.05) frequencies (Supplementary Fig. 4). The sub-study focusing on the combined group of WMU and COI patients reached a very decent power of >80 % to identify the allele associations of common variants at the OR > 1.5 and rare variants at the OR > 2 (Supplementary Fig. 4 C).

The Hardy–Weinberg equilibrium analysis indicated a deviation from the equilibrium in the case of rs2279017 (*XPC*) at *p* = 4.48E−04 and rs11615 (*ERCC1*) *p* = 4.78E−02, only in the group of CRC affected individuals collected and genotyped at WMU (Supplementary Table 4 in comparison with Supplementary Table 5 and Table 6).

The single marker, allelic association analysis performed in the whole WMU cohort indicated association of rs1208 (*NAT2*) minor allele G at OR 1.75 (1.18–2.6); *p* = 2.72E−02 (Supplementary Table 7). A very similar result was obtained for the WMU cohort individuals 50 years of age and above (Supplementary Table 8). This association showed a slight tendency of gender specificity being more pronounced in the female group of individuals after 50 years of age.

In contrast, a similar allelic association analysis performed in the COI cohort showed an association with CRC neither in the whole group nor in the group of individuals above 50 years of age as well as combined groups (Supplementary Table 9, Table 10 and Table 11).

Interestingly for the combined cohort of WMU and COI patients, 50 years of age or above, we identified an independent (not seen in any of the individual cohorts) association of the marker (rs1048943) found in the CYP1A1 gene OR 2.05 (1.29–3.28); *p* = 1.25E−02 (Supplementary Table 12 A). The association showed quite pronounced gender specificity being stronger in the female group of CRC patients OR 2.72 (1.43–5.14); *p* = 1.14E−02 (Supplementary Table 12 C).

The logistic regression model analysis confirmed the results of the allelic one also indicating an important influence of gender for the overall CRC susceptibility, with males being at somewhat greater risk of CRC OR 1.48 (1.11–1.98); *p* = 4.55E−02 (Supplementary Table 15 A). This effect was especially strong in the WMU cohort OR 3.18 (1.73–5.84); *p* = 1.19E−03 (Supplementary Table 13 A). However, in COI patients group, this effect was not seen (Supplementary Table 14). The receiver operating characteristic (ROC) plots computed based on the logistic regression additive model showed that the cumulative effects of selected markers were a moderately strong predictor of increased risk of CRC scoring AUC (area under the curve) parameter values of 0.709, 0.576 and 0.586 for the WMU, COI and combined cohorts, respectively (Supplementary Fig. 5). In general, the indicator performance was observed to be slightly better for the cohorts 50 years of age or above.

## Discussion

In Poland, an upward trend of sporadic colorectal cancer (CRC), among both male and female individuals, is observed. It has been estimated that during forthcoming years (till 2025), the number of cases will almost double [19]. Annually, approximately 14,600 of new cases of the disease are diagnosed. Average rate of 5-year survival remains at a similar level for both sexes and approximates 30 percent. It is estimated that the mortality from CRC in the next 20 years will double, especially in patients over 65 years of age [31].

The main risk factors for disease are age (about 65 % of elderly patients), gender (1,4:1 male to female ratio), inflammatory bowel disease, family history of CRC, individual susceptibility to cancer modulated by low penetration gene polymorphisms and external factors such as diet and lifestyle [32].

In this study, we aimed to assess the role of chosen polymorphisms as a risk factors in carcinogenesis of colon. Only one of the studied SNPs, the CYP1A1 Ile462Val variant (minor allele), emerged as a single-putative risk allele in sporadic colorectal cancer patients especially for female over 50 years old.

CYP1A1 gene is located on a long arm of chromosome 15 (15q24.1) and belongs to the cytochrome P450 enzymes family which participate in the first phase of xenobiotic metabolism. Main substrates of CYP1A1 include many environmental carcinogens such as alkaloids or heterocyclic aromatic amines. Hormones (e.g., estrone, testosterone) and some drugs are also metabolized during first phase via CYP1A1 [33]. Polymorphisms present in CYP1A1 gene may influence effectiveness of the metabolism of environmental carcinogens. The A>G transition in codon 462 (exon 7) leads to a substitution of isoleucine to valine (Ile462Val). In consequence, the effectiveness of the enzyme is increased and thereby higher amount of carcinogenic active molecules, especially polycyclic aromatic hydrocarbons PAHs, may be involved in carcinogenesis of colorectal mucosa [33].

The assessment of Ile462Val polymorphism has been extensively investigated in various cancers such as: endometrial, lung, cervical, oral, head and neck, ovarian, gastric, breast and colorectal [16–28], as well as on healthy population, e.g., Polish from Silesian region [34]. However, the role of this polymorphism remains ambiguous and not fully explored because of the low frequency of the minor allele especially in Caucasians.

Recently, two meta-analyses, done by Jin (2011) and Zheng (2012), concerning CYP1A1 Ile462Val polymorphism and its role in colorectal cancer risk have been published [25, 26]. Jin et al. took into consideration thirteen case–control studies consisting of 5,336 CRC cases and 6,226 controls. They concluded that CYP1A1 Ile462Val polymorphism may contribute to colorectal cancer risk in Asians as well as Europeans [25].

Zheng et al. investigated fourteen studies including 6,654 CRC cases and 7,895 controls. They found a statistically significant relationship between CYP1A1 Ile462Val minor allele and increased risk of colorectal cancer (recessive model: OR 1.45, 95 % CI 1.16–1.81) [26].

Moreover, our analysis clearly showed that the design of the study especially size of the group (number of participants) plays a key role. The effect of *CYP1A* Ile462Val polymorphism emerged only in combined groups (in smaller it was not seen) what allows us to propose a thesis that before choosing the sample size the power of the statistical test should be assessed; otherwise, significance of rare minor alleles may be overlooked.

Hence, we imply the CYP1A1 Ile462Val polymorphism should be taken under consideration for next larger scale studies to support/reject its role in CRC tumorigenesis in Polish population.

## Electronic supplementary material

Below is the link to the electronic supplementary material.

Supplementary material 1 (DOCX 209 kb)

Supplementary material 2 (DOCX 223 kb)

Supplementary material 3 (DOCX 218 kb)

Supplementary material 4 (DOCX 51 kb)

Supplementary material 5 (DOCX 242 kb)

Supplementary material 6 (DOCX 20 kb)

Supplementary material 7 (DOCX 21 kb)

Supplementary material 8 (DOCX 22 kb)

Supplementary material 9 (DOCX 22 kb)

Supplementary material 10 (DOCX 21 kb)

Supplementary material 11 (DOCX 21 kb)

Supplementary material 12 (DOCX 21 kb)

Supplementary material 13 (DOCX 20 kb)

Supplementary material 14 (DOCX 20 kb)

Supplementary material 15 (DOCX 20 kb)

Supplementary material 16 (DOCX 20 kb)

Supplementary material 17 (DOCX 20 kb)

Supplementary material 18 (DOCX 22 kb)

Supplementary material 19 (DOCX 23 kb)

Supplementary material 20 (DOCX 21 kb)

## References

[1] Khan N, Afaq F, Mukhtar H (2010). Lifestyle as risk factor for cancer: evidence from human studies. Cancer Lett.

[2] Ahmed FE (2006). Gene-gene, gene-environment & multiple interactions in colorectal cancer. J Environ Sci Health C Environ Carcinog Ecotoxicol Rev.

[3] Zhang K, Civan J, Mukherjee S, Patel F, Yang H (2014). Genetic variations in colorectal cancer risk and clinical outcome. World J Gastroenterol.

[4] Ni M, Zhang WZ, Qiu JR, Liu F, Li M, Zhang YJ, Liu Q, Bai J (2014). Association of ERCC1 and ERCC2 polymorphisms with colorectal cancer risk in a Chinese population. Sci Rep.

[5] He J, Shi TY, Zhu ML, Wang MY, Li QX, Wei QY (2013). Associations of Lys939Gln and Ala499Val polymorphisms of the XPC gene with cancer susceptibility: a meta-analysis. Int J Cancer.

[6] Liu L, Miao L, Ji G, Qiang F, Liu Z, Fan Z (2013). Association between XRCC1 and XRCC3 polymorphisms and colorectal cancer risk: a meta-analysis of 23 case-control studies. Mol Biol Rep.

[7] Liu H, Fu ZX, Wang CY, Qian J, Xing L, Liu YW (2012). A meta-analysis of the relationship between NAT2 polymorphism and colorectal cancer susceptibility. Medicina (Kaunas).

[8] Laczmanska I, Gil J, Karpinski P, Stembalska A, Kozlowska J, Busza H, Trusewicz A, Pesz K, Ramsey D, Schlade-Bartusiak K, Blin N, Sasiadek MM (2006). Influence of polymorphisms in xenobiotic-metabolizing genes and DNA-repair genes on diepoxybutane-induced SCE frequency. Environ Mol Mutagen.

[9] Koutros S, Andreotti G, Berndt SI, Hughes Barry K, Lubin JH, Hoppin JA, Kamel F, Sandler DP, Burdette LA, Yuenger J, Yeager M, Alavanja MC, Freeman LE (2011). Xenobiotic-metabolizing gene variants, pesticide use, and the risk of prostate cancer. Pharmacogenet Genomics.

[10] Hussein AG, Pasha HF, El-Shahat HM, Gad DM, Toam MM (2014). CYP1A1 gene polymorphisms and smoking status as modifier factors for lung cancer risk. Gene.

[11] Shin A, Kang D, Choi JY, Lee KM, Park SK, Noh DY, Ahn SH (2007). Yoo KY Cytochrome P450 1A1 (CYP1A1) polymorphisms and breast cancer risk in Korean women. Exp Mol Med.

[12] Sachse C, Smith G, Wilkie MJ, Barrett JH, Waxman R, Sullivan F, Forman D, Bishop DT, Wolf CR (2002). Colorectal Cancer Study Group. A pharmacogenetic study to investigate the role of dietary carcinogens in the etiology of colorectal cancer. Carcinogenesis.

[13] Przybylowska K, Kabzinski J, Sygut A, Dziki L, Dziki A, Majsterek I (2013). An association selected polymorphisms of XRCC1, OGG1 and MUTYH gene and the level of efficiency oxidative DNA damage repair with a risk of colorectal cancer. Mutat Res.

[14] Menck CF, Munford V (2014). DNA repair diseases: what do they tell us about cancer and aging?. Genet Mol Biol.

[15] Aze A, Zhou JC, Costa A, Costanzo V (2013). DNA replication and homologous recombination factors: acting together to maintain genome stability. Chromosoma.

[16] Gil J, Ramsey D, Stembalska A, Karpinski P, Pesz KA, Laczmanska I, Leszczynski P, Grzebieniak Z, Sasiadek MM (2012). The C/A polymorphism in intron 11 of the XPC gene plays a crucial role in the modulation of an individual’s susceptibility to sporadic colorectal cancer. Mol Biol Rep.

[17] Zhuo W, Zhang L, Zhu B, Qiu Z, Chen Z (2012). Association between CYP1A1 Ile462Val variation and acute leukemia risk: meta-analyses including 2164 cases and 4160 controls. PLoS One.

[18] Ji YN, Wang Q (2012). Suo LJ (2012) CYP1A1 Ile462Val polymorphism contributes to lung cancer susceptibility among lung squamous carcinoma and smokers: a meta-analysis. PLoS One.

[19] Yang S, Jia C, Zhu H, Han S (2012). CYP1A1 Ile462Val polymorphism and cervical cancer: evidence from a meta-analysis. Tumour Biol.

[20] Zhuo X, Zhao H, Chang A, Ye H, Zhou Y, Song Y, Tan Y (2012). Cytochrome P450 1A1 Ile462Val polymorphism and oral carcinoma risk: an updated meta-analysis including 1,515 cases and 2,233 controls. Tumour Biol.

[21] Sergentanis TN, Economopoulos KP, Choussein S, Vlahos NF (2012). Cytochrome P450 1A1 (CYP1A1) gene polymorphisms and ovarian cancer risk: a meta-analysis. Mol Biol Rep.

[22] Reszka E, Czekaj P, Adamska J, Wasowicz W (2008). Relevance of glutathione S-transferase M1 and cytochrome P450 1A1 genetic polymorphisms to the development of head and neck cancers. Clin Chem Lab Med.

[23] Guo R, Guo X (2012). Quantitative assessment of the associations between CYP1A1 polymorphisms and gastric cancer risk. Tumour Biol.

[24] Shin A, Kang D, Choi JY, Lee KM, Park SK, Noh DY, Ahn SH (2007). Yoo KY Cytochrome P450 1A1 (CYP1A1) polymorphisms and breast cancer risk in Korean women. Exp Mol Med.

[25] Jin JQ, Hu YY, Niu YM, Yang GL, Wu YY, Leng WD, Xia LY (2011). CYP1A1 Ile462Val polymorphism contributes to colorectal cancer risk: a meta-analysis. World J Gastroenterol.

[26] Zheng Y, Wang JJ, Sun L, Li HL (2012). Association between CYP1A1 polymorphism and colorectal cancer risk: a meta-analysis. Mol Biol Rep.

[27] Mrozikiewicz PM, Grześkowiak E, Seremak-Mrozikiewicz A, Bogacz A, Barlik M, Semczuk A, Bartkowiak-Wieczorek J, Drews K (2011). Importance of CYP1A1 polymorphism and its transcriptional regulation in ovarian and endometrial cancer. Ginekol Pol.

[28] Ociepa-Zawal M, Rubis B, Filas V, Breborowicz J, Trzeciak WH (2009). Studies on CYP1A1, CYP1B1 and CYP3A4 gene polymorphisms in breast cancer patients. Ginekol Pol.

[29] Dupont WD, Plummer WD (1990). Power and Sample Size Calculations: a review and computer program. Control Clin Trials.

[30] Virasakdi C (2012) Epidemiological calculator. R package version 2.15.1.0.

[31] Ditkowska J, Wojciechowska U, Zatonski W (2009) Prediction of cancer incidence and mortality in Poland up to the year 2025. Report published within “Cancer Registration” p. 23–28.

[32] Aiello M, Vella N, Cannavò C, Scalisi A, Spandidos DA, Toffoli G, Buonadonna A, Libra M, Stivala F (2011). Role of genetic polymorphisms and mutations in colorectal cancer therapy (Review). Mol Med Rep.

[33] Akiyama TE, Gonzalez FJ (2003). Regulation of P450 genes by liver-enriched transcription factors and nuclear receptors. Biochim Biophys Acta.

[34] Motykiewicz G, Michalska J, Pendzich J, Małusecka E, Strózyk M, Kalinowska E, Butkiewicz D, Mielzyńska D, Midro A (1998). Santella RM, Chorazy M A molecular epidemiology study in women from Upper Silesia Poland. Toxicol Lett.

