# Supplementary material for: CYP1A1 Ile462Val polymorphism and colorectal cancer risk in Polish patients
Source: Med Oncol. 2014 Jun 18;31(7):72. doi: 10.1007/s12032-014-0072-y (PMC4079939; doi:10.1007/s12032-014-0072-y)
Supplement: Supplementary file 1 — Supplementary material 1 (DOCX 209 kb) [file 12032_2014_72_MOESM1_ESM.docx]

Supplementary Figure 1 Patient and control groups age distribution within Wroclaw Medical University (WMU) cohort.
