# Supplementary material for: CYP1A1 Ile462Val polymorphism and colorectal cancer risk in Polish patients
Source: Med Oncol. 2014 Jun 18;31(7):72. doi: 10.1007/s12032-014-0072-y (PMC4079939; doi:10.1007/s12032-014-0072-y)
Supplement: Supplementary file 2 — Supplementary material 2 (DOCX 223 kb) [file 12032_2014_72_MOESM2_ESM.docx]

Supplementary Figure 2 Patient and control groups age distribution within Warsaw Center of Oncology – Institute (COI) cohort.
