# Supplementary material for: CYP1A1 Ile462Val polymorphism and colorectal cancer risk in Polish patients
Source: Med Oncol. 2014 Jun 18;31(7):72. doi: 10.1007/s12032-014-0072-y (PMC4079939; doi:10.1007/s12032-014-0072-y)
Supplement: Supplementary file 3 — Supplementary material 3 (DOCX 218 kb) [file 12032_2014_72_MOESM3_ESM.docx]

Supplementary Figure 3 Patient and control groups age distribution within the combined Warsaw Center of Oncology – Institute (COI) and Wroclaw Medical University (WMU) cohort.
