# Supplementary material for: CYP1A1 Ile462Val polymorphism and colorectal cancer risk in Polish patients
Source: Med Oncol. 2014 Jun 18;31(7):72. doi: 10.1007/s12032-014-0072-y (PMC4079939; doi:10.1007/s12032-014-0072-y)
Supplement: Supplementary file 4 — Supplementary material 4 (DOCX 51 kb) [file 12032_2014_72_MOESM4_ESM.docx]

Supplementary Figure 4. Statistical power of the study design including all studied individuals. p0: frequency of the studied allele in the control group of subjects. Wroclaw Medical University (WMU) (A); Warsaw Center of Oncology – Institute (COI) (B); Combined Warsaw Center of Oncology – Institute (COI) and Wroclaw Medical University (WMU) patient group (C)

1. B)

C)
