# Supplementary material for: CYP1A1 Ile462Val polymorphism and colorectal cancer risk in Polish patients
Source: Med Oncol. 2014 Jun 18;31(7):72. doi: 10.1007/s12032-014-0072-y (PMC4079939; doi:10.1007/s12032-014-0072-y)
Supplement: Supplementary file 5 — Supplementary material 5 (DOCX 242 kb) [file 12032_2014_72_MOESM5_ESM.docx]

Supplementary Figure 5. Receiver operating characteristic (ROC) plot based on the multivariate logistic regression model. The ROC plots, as well as the respective AUC (area under the curve) parameters indicate a slightly better predictive performance of the studied marker set in the group of individuals 50 years of age or above. Wroclaw Medical University (WMU) cohort (A); Warsaw Center of Oncology – Institute (COI) cohort (B); Combined Warsaw Center of Oncology – Institute (COI) and Wroclaw Medical University (WMU) cohort (C).

A B

C
