# Supplementary material for: CYP1A1 Ile462Val polymorphism and colorectal cancer risk in Polish patients
Source: Med Oncol. 2014 Jun 18;31(7):72. doi: 10.1007/s12032-014-0072-y (PMC4079939; doi:10.1007/s12032-014-0072-y)
Supplement: Supplementary file 6 — Supplementary material 6 (DOCX 20 kb) [file 12032_2014_72_MOESM6_ESM.docx]

Supplementary Table 1. Wroclaw Medical University (WMU) patient group size and age statistics. Whole cohort (A); subjects 50 years of age or above (B).

A)

|  |  | N | min | max | median | mean | SD |
| --- | --- | --- | --- | --- | --- | --- | --- |
| case | all | 110 | 33 | 87 | 66.00 | 65.64 | 10.10 |
|  | female | 48 | 45 | 87 | 67.00 | 66.48 | 9.36 |
|  | male | 62 | 33 | 84 | 66.00 | 64.98 | 10.66 |
| control | all | 100 | 58 | 97 | 75.00 | 74.89 | 7.63 |
|  | female | 70 | 60 | 97 | 76.50 | 76.04 | 7.54 |
|  | male | 30 | 58 | 87 | 71.50 | 72.20 | 7.26 |

B)

|  |  | N | min | max | median | mean | SD |
| --- | --- | --- | --- | --- | --- | --- | --- |
| case | all | 101 | 53 | 87 | 67.00 | 67.50 | 8.12 |
|  | female | 45 | 53 | 87 | 68.00 | 67.76 | 8.16 |
|  | male | 65 | 53 | 84 | 66.50 | 67.29 | 8.16 |
| control | all | 100 | 58 | 97 | 75.00 | 74.89 | 7.62 |
|  | female | 70 | 60 | 97 | 76.50 | 76.04 | 7.54 |
|  | male | 30 | 58 | 87 | 71.50 | 72.20 | 7.26 |
