# Supplementary material for: CYP1A1 Ile462Val polymorphism and colorectal cancer risk in Polish patients
Source: Med Oncol. 2014 Jun 18;31(7):72. doi: 10.1007/s12032-014-0072-y (PMC4079939; doi:10.1007/s12032-014-0072-y)
Supplement: Supplementary file 7 — Supplementary material 7 (DOCX 21 kb) [file 12032_2014_72_MOESM7_ESM.docx]

Supplementary Table 10. Marker allele association for the Warsaw Center of Oncology – Institute (COI) patients 50 years of age or above. All (A); females (B); males (C). Minor allele (A1); major allele (A2).

A)

| **SNP** | **Chr.** | **Pos. NCBI (hg19)** | **Gene** | **A1** | **A1_Affected** | **A1_Unaffected** | **A2** | **OR (95% CI)** | **p-value (Fisher ex. test)** | **p-value _cor._ Bonf.** | **p-value _cor._ BH** |
| --- | --- | --- | --- | --- | --- | --- | --- | --- | --- | --- | --- |
| rs2279017 | 3 | 14190237 | XPC | T | 0.36 | 0.41 | G | 0.81 (0.61-1.09) | 1.66E-01 | 8.31E-01 | 3.39E-01 |
| rs1208 | 8 | 18258316 | NAT2 | G | 0.44 | 0.41 | A | 1.13 (0.85-1.5) | 4.29E-01 | 1.00E+00 | 4.29E-01 |
| rs861539 | 14 | 104165753 | XRCC3 | A | 0.37 | 0.32 | G | 1.21 (0.9-1.62) | 2.04E-01 | 1.00E+00 | 3.39E-01 |
| rs1048943 | 15 | 75012985 | CYP1A1 | C | 0.05 | 0.04 | T | 1.51 (0.77-2.96) | 2.84E-01 | 1.00E+00 | 3.55E-01 |
| rs11615 | 19 | 45923653 | ERCC1 | G | 0.40 | 0.33 | A | 1.34 (1-1.79) | 5.28E-02 | 2.64E-01 | 2.64E-01 |

B)

| **SNP** | **Chr.** | **Pos. NCBI (hg19)** | **Gene** | **A1** | **A1_Affected** | **A1_Unaffected** | **A2** | **OR (95% CI)** | **p-value (Fisher ex. test)** | **p-value _cor._ Bonf.** | **p-value _cor._ BH** |
| --- | --- | --- | --- | --- | --- | --- | --- | --- | --- | --- | --- |
| rs2279017 | 3 | 14190237 | XPC | T | 0.34 | 0.43 | G | 0.69 (0.49-0.98) | 4.36E-02 | 2.18E-01 | 1.15E-01 |
| rs1208 | 8 | 18258316 | NAT2 | G | 0.45 | 0.41 | A | 1.19 (0.85-1.68) | 3.40E-01 | 1.00E+00 | 4.64E-01 |
| rs861539 | 14 | 104165753 | XRCC3 | A | 0.34 | 0.34 | G | 0.98 (0.69-1.39) | 9.28E-01 | 1.00E+00 | 9.28E-01 |
| rs1048943 | 15 | 75012985 | CYP1A1 | C | 0.06 | 0.03 | T | 2.38 (1.05-5.42) | 4.60E-02 | 2.30E-01 | 1.15E-01 |
| rs11615 | 19 | 45923653 | ERCC1 | G | 0.39 | 0.35 | A | 1.18 (0.83-1.67) | 3.71E-01 | 1.00E+00 | 4.64E-01 |

C)

| **SNP** | **Chr.** | **Pos. NCBI (hg19)** | **Gene** | **A1** | **A1_Affected** | **A1_Unaffected** | **A2** | **OR (95% CI)** | **p-value (Fisher ex. test)** | **p-value _cor._ Bonf.** | **p-value _cor._ BH** |
| --- | --- | --- | --- | --- | --- | --- | --- | --- | --- | --- | --- |
| rs2279017 | 3 | 14190237 | XPC | T | 0.41 | 0.37 | G | 1.17 (0.7-1.96) | 5.99E-01 | 1.00E+00 | 5.99E-01 |
| rs1208 | 8 | 18258316 | NAT2 | G | 0.42 | 0.42 | A | 1.01 (0.61-1.67) | 1.00E+00 | NA | NA |
| rs861539 | 14 | 104165753 | XRCC3 | A | 0.43 | 0.28 | G | 1.93 (1.14-3.26) | 1.55E-02 | 6.18E-02 | 6.18E-02 |
| rs1048943 | 15 | 75012985 | CYP1A1 | C | 0.03 | 0.05 | T | 0.55 (0.14-2.07) | 5.44E-01 | 1.00E+00 | 5.99E-01 |
| rs11615 | 19 | 45923653 | ERCC1 | G | 0.41 | 0.28 | A | 1.82 (1.08-3.06) | 3.12E-02 | 1.25E-01 | 6.24E-02 |
