# Supplementary material for: CYP1A1 Ile462Val polymorphism and colorectal cancer risk in Polish patients
Source: Med Oncol. 2014 Jun 18;31(7):72. doi: 10.1007/s12032-014-0072-y (PMC4079939; doi:10.1007/s12032-014-0072-y)
Supplement: Supplementary file 8 — Supplementary material 8 (DOCX 22 kb) [file 12032_2014_72_MOESM8_ESM.docx]

Supplementary Table 11. Marker allele association for the combined Warsaw Center of Oncology – Institute (COI) and Wroclaw Medical University (WMU) cohort. All (A); females (B); males (C). Minor allele (A1); major allele (A2).

A)

| **SNP** | **Chr.** | **Pos. NCBI Build 37** | **Gene** | **A1** | **A1_Affected** | **A1_Unaffected** | **A2** | **OR (95% CI)** | **p-value (Fisher ex. test)** | **p-value _cor._ Bonf.** | **p-value _cor._ BH** |
| --- | --- | --- | --- | --- | --- | --- | --- | --- | --- | --- | --- |
| rs2279017 | 3 | 14190237 | XPC | T | 0.40 | 0.41 | G | 0.97 (0.8-1.17) | 7.68E-01 | 1.00E+00 | 7.68E-01 |
| rs1208 | 8 | 18258316 | NAT2 | G | 0.42 | 0.39 | A | 1.13 (0.93-1.37) | 2.21E-01 | 1.00E+00 | 3.69E-01 |
| rs861539 | 14 | 104165753 | XRCC3 | A | 0.35 | 0.33 | G | 1.11 (0.91-1.35) | 3.35E-01 | 1.00E+00 | 4.19E-01 |
| rs1048943 | 15 | 75012985 | CYP1A1 | C | 0.07 | 0.04 | T | 1.7 (1.11-2.61) | 1.62E-02 | 8.08E-02 | 8.08E-02 |
| rs11615 | 19 | 45923653 | ERCC1 | G | 0.39 | 0.35 | A | 1.18 (0.97-1.43) | 1.11E-01 | 5.54E-01 | 2.77E-01 |

B)

| **SNP** | **Chr.** | **Pos. NCBI Build 37** | **Gene** | **A1** | **A1_Affected** | **A1_Unaffected** | **A2** | **OR (95% CI)** | **p-value (Fisher ex. test)** | **p-value _cor._ Bonf.** | **p-value _cor._ BH** |
| --- | --- | --- | --- | --- | --- | --- | --- | --- | --- | --- | --- |
| rs2279017 | 3 | 14190237 | XPC | T | 0.38 | 0.43 | G | 0.83 (0.65-1.05) | 1.30E-01 | 6.48E-01 | 3.24E-01 |
| rs1208 | 8 | 18258316 | NAT2 | G | 0.42 | 0.40 | A | 1.1 (0.87-1.4) | 4.33E-01 | 1.00E+00 | 5.74E-01 |
| rs861539 | 14 | 104165753 | XRCC3 | A | 0.34 | 0.35 | G | 0.98 (0.76-1.25) | 8.51E-01 | 1.00E+00 | 8.51E-01 |
| rs1048943 | 15 | 75012985 | CYP1A1 | C | 0.06 | 0.03 | T | 2.07 (1.14-3.73) | 1.56E-02 | 7.80E-02 | 7.80E-02 |
| rs11615 | 19 | 45923653 | ERCC1 | G | 0.38 | 0.36 | A | 1.1 (0.87-1.4) | 4.59E-01 | 1.00E+00 | 5.74E-01 |

C)

| **SNP** | **Chr.** | **Pos. NCBI Build 37** | **Gene** | **A1** | **A1_Affected** | **A1_Unaffected** | **A2** | **OR (95% CI)** | **p-value (Fisher ex. test)** | **p-value _cor._ Bonf.** | **p-value _cor._ BH** |
| --- | --- | --- | --- | --- | --- | --- | --- | --- | --- | --- | --- |
| rs2279017 | 3 | 14190237 | XPC | T | 0.4258 | 0.3602 | G | 1.32 (0.94-1.85) | 1.25E-01 | 6.23E-01 | 2.08E-01 |
| rs1208 | 8 | 18258316 | NAT2 | G | 0.4212 | 0.3782 | A | 1.2 (0.86-1.67) | 3.10E-01 | 1.00E+00 | 3.87E-01 |
| rs861539 | 14 | 104165753 | XRCC3 | A | 0.3743 | 0.2941 | G | 1.44 (1.01-2.04) | 4.41E-02 | 2.20E-01 | 2.08E-01 |
| rs1048943 | 15 | 75012985 | CYP1A1 | C | 0.08021 | 0.0678 | T | 1.2 (0.64-2.25) | 6.39E-01 | 1.00E+00 | 6.39E-01 |
| rs11615 | 19 | 45923653 | ERCC1 | G | 0.3978 | 0.3292 | A | 1.35 (0.96-1.89) | 8.75E-02 | 4.37E-01 | 2.08E-01 |
