# Supplementary material for: CYP1A1 Ile462Val polymorphism and colorectal cancer risk in Polish patients
Source: Med Oncol. 2014 Jun 18;31(7):72. doi: 10.1007/s12032-014-0072-y (PMC4079939; doi:10.1007/s12032-014-0072-y)
Supplement: Supplementary file 9 — Supplementary material 9 (DOCX 22 kb) [file 12032_2014_72_MOESM9_ESM.docx]

Supplementary Table 12. Marker allele association for the combined Warsaw Center of Oncology – Institute (COI) and Wroclaw Medical University (WMU) patients 50 years of age or above. All (A); females (B); males (C). Minor allele (A1); major allele (A2).

A)

| **SNP** | **Chr.** | **Pos. NCBI Build 37** | **Gene** | **A1** | **A1_Affected** | **A1_Unaffected** | **A2** | **OR (95% CI)** | **p-value (Fisher ex. test)** | **p-value _cor._ Bonf.** | **p-value _cor._ BH** |
| --- | --- | --- | --- | --- | --- | --- | --- | --- | --- | --- | --- |
| rs2279017 | 3 | 14190237 | XPC | T | 0.41 | 0.41 | G | 1.01 (0.8-1.27) | 9.53E-01 | 1.00E+00 | 9.53E-01 |
| rs1208 | 8 | 18258316 | NAT2 | G | 0.46 | 0.39 | A | 1.31 (1.05-1.65) | 2.02E-02 | 1.01E-01 | 5.04E-02 |
| rs861539 | 14 | 104165753 | XRCC3 | A | 0.35 | 0.32 | G | 1.14 (0.9-1.45) | 3.01E-01 | 1.00E+00 | 3.76E-01 |
| rs1048943 | 15 | 75012985 | CYP1A1 | C | 0.08 | 0.04 | T | 2.05 (1.29-3.28) | 2.49E-03 | 1.25E-02 | 1.25E-02 |
| rs11615 | 19 | 45923653 | ERCC1 | G | 0.40 | 0.35 | A | 1.24 (0.99-1.57) | 7.42E-02 | 3.71E-01 | 1.24E-01 |

B)

| **SNP** | **Chr.** | **Pos. NCBI Build 37** | **Gene** | **A1** | **A1_Affected** | **A1_Unaffected** | **A2** | **OR (95% CI)** | **p-value (Fisher ex. test)** | **p-value _cor._ Bonf.** | **p-value _cor._ BH** |
| --- | --- | --- | --- | --- | --- | --- | --- | --- | --- | --- | --- |
| rs2279017 | 3 | 14190237 | XPC | T | 0.38 | 0.42 | G | 0.85 (0.63-1.14) | 3.02E-01 | 1.00E+00 | 5.03E-01 |
| rs1208 | 8 | 18258316 | NAT2 | G | 0.46 | 0.40 | A | 1.29 (0.97-1.72) | 9.19E-02 | 4.59E-01 | 2.30E-01 |
| rs861539 | 14 | 104165753 | XRCC3 | A | 0.32 | 0.34 | G | 0.91 (0.67-1.23) | 5.91E-01 | 1.00E+00 | 5.91E-01 |
| rs1048943 | 15 | 75012985 | CYP1A1 | C | 0.08 | 0.03 | T | 2.72 (1.43-5.14) | 2.27E-03 | 1.14E-02 | 1.14E-02 |
| rs11615 | 19 | 45923653 | ERCC1 | G | 0.38 | 0.36 | A | 1.1 (0.82-1.48) | 5.46E-01 | 1.00E+00 | 5.91E-01 |

C)

| **SNP** | **Chr.** | **Pos. NCBI Build 37** | **Gene** | **A1** | **A1_Affected** | **A1_Unaffected** | **A2** | **OR (95% CI)** | **p-value (Fisher ex. test)** | **p-value _cor._ Bonf.** | **p-value _cor._ BH** |
| --- | --- | --- | --- | --- | --- | --- | --- | --- | --- | --- | --- |
| rs2279017 | 3 | 14190237 | XPC | T | 0.45 | 0.37 | G | 1.39 (0.95-2.05) | 9.65E-02 | 4.82E-01 | 1.50E-01 |
| rs1208 | 8 | 18258316 | NAT2 | G | 0.46 | 0.38 | A | 1.37 (0.94-2.01) | 1.20E-01 | 6.00E-01 | 1.50E-01 |
| rs861539 | 14 | 104165753 | XRCC3 | A | 0.40 | 0.28 | G | 1.69 (1.13-2.53) | 1.12E-02 | 5.59E-02 | 5.59E-02 |
| rs1048943 | 15 | 75012985 | CYP1A1 | C | 0.09 | 0.07 | T | 1.29 (0.65-2.58) | 4.86E-01 | 1.00E+00 | 4.86E-01 |
| rs11615 | 19 | 45923653 | ERCC1 | G | 0.42 | 0.32 | A | 1.54 (1.04-2.26) | 3.81E-02 | 1.91E-01 | 9.53E-02 |
