# Supplementary material for: CYP1A1 Ile462Val polymorphism and colorectal cancer risk in Polish patients
Source: Med Oncol. 2014 Jun 18;31(7):72. doi: 10.1007/s12032-014-0072-y (PMC4079939; doi:10.1007/s12032-014-0072-y)
Supplement: Supplementary file 10 — Supplementary material 10 (DOCX 21 kb) [file 12032_2014_72_MOESM10_ESM.docx]

Supplementary Table 13. Multivariate logistic regression considering the additive model of gene action. Wroclaw Medical University (WMU) patients. Whole cohort (A); subjects 50 years of age or above (B).

A)

| **Factor** | **Chr.** | **Pos. NCBI Build 37** | **Gene** | **OR (95% CI)** | **p-value** | **p-value _cor._ Bonf.** | **p-value _cor._ BH** |
| --- | --- | --- | --- | --- | --- | --- | --- |
| sex (index = female) |  |  |  | 3.18 (1.73-5.84) | 1.98E-04 | 1.19E-03 | 1.19E-03 |
| rs2279017 | 3 | 14190237 | XPC | 1.53 (0.95-2.46) | 7.80E-02 | 4.68E-01 | 1.56E-01 |
| rs1208 | 8 | 18258316 | NAT2 | 0.52 (0.33-0.81) | 4.07E-03 | 2.44E-02 | 1.22E-02 |
| rs861539 | 14 | 104165753 | XRCC3 | 1.06 (0.67-1.67) | 8.11E-01 | 1.00E+00 | 8.30E-01 |
| rs1048943 | 15 | 75012985 | CYP1A1 | 1.81 (0.87-3.75) | 1.11E-01 | 6.65E-01 | 1.66E-01 |
| rs11615 | 19 | 45923653 | ERCC1 | 0.95 (0.6-1.5) | 8.30E-01 | 1.00E+00 | 8.30E-01 |

B)

| **Factor** | **Chr.** | **Pos. NCBI Build 37** | **Gene** | **OR (95% CI)** | **p-value** | **p-value _cor._ Bonf.** | **p-value _cor._ BH** |
| --- | --- | --- | --- | --- | --- | --- | --- |
| sex (index = female) |  |  |  | 3.06 (1.64-5.7) | 4.36E-04 | 2.62E-03 | 2.62E-03 |
| rs2279017 | 3 | 14190237 | XPC | 1.62 (0.99-2.63) | 5.33E-02 | 3.20E-01 | 1.07E-01 |
| rs1208 | 8 | 18258316 | NAT2 | 0.5 (0.32-0.79) | 2.99E-03 | 1.79E-02 | 8.96E-03 |
| rs861539 | 14 | 104165753 | XRCC3 | 1.02 (0.64-1.63) | 9.18E-01 | 1.00E+00 | 9.18E-01 |
| rs1048943 | 15 | 75012985 | CYP1A1 | 1.81 (0.87-3.78) | 1.14E-01 | 6.83E-01 | 1.71E-01 |
| rs11615 | 19 | 45923653 | ERCC1 | 0.97 (0.61-1.55) | 9.06E-01 | 1.00E+00 | 9.18E-01 |
