# Supplementary material for: CYP1A1 Ile462Val polymorphism and colorectal cancer risk in Polish patients
Source: Med Oncol. 2014 Jun 18;31(7):72. doi: 10.1007/s12032-014-0072-y (PMC4079939; doi:10.1007/s12032-014-0072-y)
Supplement: Supplementary file 11 — Supplementary material 11 (DOCX 21 kb) [file 12032_2014_72_MOESM11_ESM.docx]

Supplementary Table 14. Multivariate logistic regression considering the additive model of gene action. Warsaw Center of Oncology – Institute (COI) patients. Whole cohort (A); subjects 50 years of age or above (B).

A)

| **Factor** | **Chr.** | **Pos. NCBI Build 37** | **Gene** | **OR (95% CI)** | **p-value** | **p-value _cor._ Bonf.** | **p-value _cor._ BH** |
| --- | --- | --- | --- | --- | --- | --- | --- |
| sex (index = female) |  |  |  | 1.21 (0.86-1.69) | 2.76E-01 | 1.00E+00 | 3.32E-01 |
| rs2279017 | 3 | 14190237 | XPC | 0.86 (0.69-1.07) | 1.76E-01 | 1.00E+00 | 3.27E-01 |
| rs1208 | 8 | 18258316 | NAT2 | 0.95 (0.76-1.19) | 6.36E-01 | 1.00E+00 | 6.36E-01 |
| rs861539 | 14 | 104165753 | XRCC3 | 1.16 (0.92-1.47) | 2.18E-01 | 1.00E+00 | 3.27E-01 |
| rs1048943 | 15 | 75012985 | CYP1A1 | 1.53 (0.87-2.67) | 1.36E-01 | 8.19E-01 | 3.27E-01 |
| rs11615 | 19 | 45923653 | ERCC1 | 1.25 (0.99-1.58) | 5.84E-02 | 3.51E-01 | 3.27E-01 |

B)

| **Factor** | **Chr.** | **Pos. NCBI Build 37** | **Gene** | **OR (95% CI)** | **p-value** | **p-value _cor._ Bonf.** | **p-value _cor._ BH** |
| --- | --- | --- | --- | --- | --- | --- | --- |
| sex (index = female) |  |  |  | 1.15 (0.75-1.76) | 5.36E-01 | 1.00E+00 | 5.40E-01 |
| rs2279017 | 3 | 14190237 | XPC | 1.55 (0.8-2.98) | 1.92E-01 | 1.00E+00 | 3.25E-01 |
| rs1208 | 8 | 18258316 | NAT2 | 0.82 (0.62-1.09) | 1.64E-01 | 9.86E-01 | 3.25E-01 |
| rs861539 | 14 | 104165753 | XRCC3 | 1.3 (0.96-1.74) | 8.53E-02 | 5.12E-01 | 3.25E-01 |
| rs1048943 | 15 | 75012985 | CYP1A1 | 1.21 (0.89-1.63) | 2.17E-01 | 1.00E+00 | 3.25E-01 |
| rs11615 | 19 | 45923653 | ERCC1 | 1.09 (0.83-1.44) | 5.40E-01 | 1.00E+00 | 5.40E-01 |
