# Supplementary material for: CYP1A1 Ile462Val polymorphism and colorectal cancer risk in Polish patients
Source: Med Oncol. 2014 Jun 18;31(7):72. doi: 10.1007/s12032-014-0072-y (PMC4079939; doi:10.1007/s12032-014-0072-y)
Supplement: Supplementary file 12 — Supplementary material 12 (DOCX 21 kb) [file 12032_2014_72_MOESM12_ESM.docx]

Supplementary Table 15. Multivariate logistic regression considering the additive model of gene action. Combined Warsaw Center of Oncology – Institute (COI) and Wroclaw Medical University (WMU) patients. Whole cohort (A); subjects 50 years of age or above (B).

A)

| **Factor** | **Chr.** | **Pos. NCBI Build 37** | **Gene** | **OR (95% CI)** | **p-value** | **p-value _cor._ Bonf.** | **p-value _cor._ BH** |
| --- | --- | --- | --- | --- | --- | --- | --- |
| sex (index = female) |  |  |  | 1.48 (1.11-1.98) | 7.58E-03 | 4.55E-02 | 4.55E-02 |
| rs2279017 | 3 | 14190237 | XPC | 0.96 (0.79-1.16) | 6.51E-01 | 1.00E+00 | 6.51E-01 |
| rs1208 | 8 | 18258316 | NAT2 | 1.11 (0.91-1.35) | 3.18E-01 | 1.00E+00 | 3.82E-01 |
| rs861539 | 14 | 104165753 | XRCC3 | 1.15 (0.94-1.41) | 1.78E-01 | 1.00E+00 | 2.66E-01 |
| rs1048943 | 15 | 75012985 | CYP1A1 | 1.65 (1.08-2.54) | 2.18E-02 | 1.31E-01 | 6.55E-02 |
| rs11615 | 19 | 45923653 | ERCC1 | 1.17 (0.96-1.44) | 1.25E-01 | 7.51E-01 | 2.50E-01 |

B)

| **Factor** | **Chr.** | **Pos. NCBI Build 37** | **Gene** | **OR (95% CI)** | **p-value** | **p-value _cor._ Bonf.** | **p-value _cor._ BH** |
| --- | --- | --- | --- | --- | --- | --- | --- |
| sex (index = female) |  |  |  | 1.64 (1.17-2.3) | 4.15E-03 | 2.49E-02 | 2.21E-02 |
| rs2279017 | 3 | 14190237 | XPC | 1.02 (0.8-1.28) | 9.00E-01 | 1.00E+00 | 9.00E-01 |
| rs1208 | 8 | 18258316 | NAT2 | 1.3 (1.03-1.63) | 2.59E-02 | 1.55E-01 | 5.18E-02 |
| rs861539 | 14 | 104165753 | XRCC3 | 1.14 (0.89-1.46) | 2.86E-01 | 1.00E+00 | 3.43E-01 |
| rs1048943 | 15 | 75012985 | CYP1A1 | 1.9 (1.19-3.03) | 7.38E-03 | 4.43E-02 | 2.21E-02 |
| rs11615 | 19 | 45923653 | ERCC1 | 1.23 (0.97-1.57) | 9.20E-02 | 5.52E-01 | 1.38E-01 |
