# Supplementary material for: CYP1A1 Ile462Val polymorphism and colorectal cancer risk in Polish patients
Source: Med Oncol. 2014 Jun 18;31(7):72. doi: 10.1007/s12032-014-0072-y (PMC4079939; doi:10.1007/s12032-014-0072-y)
Supplement: Supplementary file 13 — Supplementary material 13 (DOCX 20 kb) [file 12032_2014_72_MOESM13_ESM.docx]

Supplementary Table 2. Warsaw Center of Oncology – Institute (COI) patient group size and age statistics. Whole cohort (A); subjects 50 years of age or above (B).

A)

|  |  | N | min | max | median | mean | SD |
| --- | --- | --- | --- | --- | --- | --- | --- |
| case | all | 368 | 21.00 | 83.00 | 47.00 | 48.85 | 12.23 |
|  | female | 243 | 22.00 | 79.00 | 48.00 | 49.16 | 12.12 |
|  | male | 125 | 21.00 | 83.00 | 47.00 | 48.26 | 12.45 |
| control | all | 304 | 41.00 | 69.00 | 59.00 | 58.35 | 4.92 |
|  | female | 213 | 41.00 | 69.00 | 59.00 | 58.22 | 4.84 |
|  | male | 91 | 43.00 | 69.00 | 60.00 | 58.64 | 5.12 |

B)

|  |  | N | min | max | median | mean | SD |
| --- | --- | --- | --- | --- | --- | --- | --- |
| case | all | 159 | 50.00 | 83.00 | 59.00 | 60.36 | 7.11 |
|  | female | 108 | 50.00 | 79.00 | 59.00 | 60.31 | 6.64 |
|  | male | 51 | 50.00 | 83.00 | 59.00 | 60.47 | 8.10 |
| control | all | 301 | 50.00 | 69.00 | 59.00 | 58.46 | 4.75 |
|  | female | 212 | 50.00 | 69.00 | 59.00 | 58.30 | 4.70 |
|  | male | 89 | 50.00 | 69.00 | 60.00 | 58.82 | 4.87 |
