# Supplementary material for: CYP1A1 Ile462Val polymorphism and colorectal cancer risk in Polish patients
Source: Med Oncol. 2014 Jun 18;31(7):72. doi: 10.1007/s12032-014-0072-y (PMC4079939; doi:10.1007/s12032-014-0072-y)
Supplement: Supplementary file 14 — Supplementary material 14 (DOCX 20 kb) [file 12032_2014_72_MOESM14_ESM.docx]

Supplementary Table 3. Combined Warsaw Center of Oncology – Institute (COI) and Wroclaw Medical University (WMU) patient group size and age statistics. Whole cohort (A); subjects 50 years of age or above (B).

A)

|  |  | N | min | max | median | mean | SD |
| --- | --- | --- | --- | --- | --- | --- | --- |
| case | all | 478 | 21 | 87 | 52.5 | 52.75 | 13.73 |
|  | female | 291 | 22 | 87 | 52 | 52.06 | 13.36 |
|  | male | 187 | 21 | 84 | 54 | 53.81 | 14.24 |
| control | all | 404 | 41 | 97 | 61 | 62.45 | 9.15 |
|  | female | 283 | 41 | 97 | 61 | 62.63 | 9.53 |
|  | male | 121 | 43 | 87 | 61 | 62.03 | 8.20 |

B)

|  |  | N | min | max | median | mean | SD |
| --- | --- | --- | --- | --- | --- | --- | --- |
| case | all | 260 | 50 | 87 | 62 | 63.13 | 8.27 |
|  | female | 153 | 50 | 87 | 61 | 62.5 | 7.87 |
|  | male | 107 | 50 | 84 | 63 | 64.04 | 8.78 |
| control | all | 401 | 56 | 97 | 61 | 62.55 | 9.06 |
|  | female | 282 | 50 | 97 | 61 | 62.71 | 9.46 |
|  | male | 119 | 50 | 87 | 61 | 62.19 | 8.04 |
