# Supplementary material for: CYP1A1 Ile462Val polymorphism and colorectal cancer risk in Polish patients
Source: Med Oncol. 2014 Jun 18;31(7):72. doi: 10.1007/s12032-014-0072-y (PMC4079939; doi:10.1007/s12032-014-0072-y)
Supplement: Supplementary file 15 — Supplementary material 15 (DOCX 20 kb) [file 12032_2014_72_MOESM15_ESM.docx]

Supplementary Table 4. Hardy-Weinberg equilibrium for the Wroclaw Medical University (WMU) cohort. Minor allele (A1); major allele (A2).

| **SNP** | **Chr.** | **Pos. NCBI Build 37** | **Gene** | **Test** | **A1** | **A2** | **GENOTYPES** | **O(HET)** | **E(HET)** | **p-value** |
| --- | --- | --- | --- | --- | --- | --- | --- | --- | --- | --- |
| rs2279017 | 3 | 14190237 | XPC | ALL | T | G | 30/123/55 | 0.59 | 0.49 | 4.92E-03 |
|  |  |  |  | AFF | T | G | 15/73/20 | 0.68 | 0.50 | 4.48E-04 |
|  |  |  |  | UNAFF | T | G | 15/50/35 | 0.50 | 0.48 | 8.35E-01 |
| rs1208 | 8 | 18258316 | NAT2 | ALL | G | A | 33/106/71 | 0.50 | 0.48 | 5.71E-01 |
|  |  |  |  | AFF | G | A | 22/60/28 | 0.55 | 0.50 | 4.43E-01 |
|  |  |  |  | UNAFF | G | A | 11/46/43 | 0.46 | 0.45 | 1.00E+00 |
| rs861539 | 14 | 104165753 | XRCC3 | ALL | A | G | 21/91/97 | 0.44 | 0.43 | 1.00E+00 |
|  |  |  |  | AFF | A | G | 8/55/46 | 0.50 | 0.44 | 1.89E-01 |
|  |  |  |  | UNAFF | A | G | 13/36/51 | 0.36 | 0.43 | 1.08E-01 |
| rs1048943 | 15 | 75012985 | CYP1A1 | ALL | C | T | 3/37/170 | 0.18 | 0.18 | 4.59E-01 |
|  |  |  |  | AFF | C | T | 2/26/82 | 0.24 | 0.24 | 1.00E+00 |
|  |  |  |  | UNAFF | C | T | 1/11/88 | 0.11 | 0.12 | 3.41E-01 |
| rs11615 | 19 | 45923653 | ERCC1 | ALL | G | A | 28/111/71 | 0.53 | 0.48 | 1.52E-01 |
|  |  |  |  | AFF | G | A | 12/63/35 | 0.57 | 0.48 | 4.78E-02 |
|  |  |  |  | UNAFF | G | A | 16/48/36 | 0.48 | 0.48 | 1.00E+00 |
