# Supplementary material for: CYP1A1 Ile462Val polymorphism and colorectal cancer risk in Polish patients
Source: Med Oncol. 2014 Jun 18;31(7):72. doi: 10.1007/s12032-014-0072-y (PMC4079939; doi:10.1007/s12032-014-0072-y)
Supplement: Supplementary file 16 — Supplementary material 16 (DOCX 20 kb) [file 12032_2014_72_MOESM16_ESM.docx]

Supplementary Table 5. Hardy-Weinberg equilibrium for the Warsaw Center of Oncology – Institute (COI) cohort. Minor allele (A1); major allele (A2).

| **SNP** | **Chr.** | **Pos. NCBI Build 37** | **Gene** | **Test** | **A1** | **A2** | **GENOTYPES** | **O(HET)** | **E(HET)** | **p-value** |
| --- | --- | --- | --- | --- | --- | --- | --- | --- | --- | --- |
| rs2279017 | 3 | 14190237 | XPC | ALL | T | G | 113/292/260 | 0.44 | 0.48 | 5.03E-02 |
|  |  |  |  | AFF | T | G | 55/159/144 | 0.44 | 0.47 | 3.12E-01 |
|  |  |  |  | UNAFF | T | G | 58/129/112 | 0.43 | 0.48 | 7.24E-02 |
| rs1208 | 8 | 18258316 | NAT2 | ALL | G | A | 115/318/237 | 0.47 | 0.48 | 6.32E-01 |
|  |  |  |  | AFF | G | A | 54/187/123 | 0.51 | 0.48 | 2.33E-01 |
|  |  |  |  | UNAFF | G | A | 59/126/113 | 0.42 | 0.48 | 3.12E-02 |
| rs861539 | 14 | 104165753 | XRCC3 | ALL | A | G | 82/307/279 | 0.46 | 0.46 | 9.33E-01 |
|  |  |  |  | AFF | A | G | 47/167/146 | 0.46 | 0.46 | 1.00E+00 |
|  |  |  |  | UNAFF | A | G | 34/134/132 | 0.45 | 0.45 | 1.00E+00 |
| rs1048943 | 15 | 75012985 | CYP1A1 | ALL | C | T | 2/54/618 | 0.08 | 0.08 | 3.50E-01 |
|  |  |  |  | AFF | C | T | 1/33/332 | 0.09 | 0.09 | 5.74E-01 |
|  |  |  |  | UNAFF | C | T | 1/18/281 | 0.06 | 0.06 | 2.79E-01 |
| rs11615 | 19 | 45923653 | ERCC1 | ALL | G | A | 90/306/272 | 0.46 | 0.46 | 8.02E-01 |
|  |  |  |  | AFF | G | A | 59/163/141 | 0.45 | 0.47 | 3.20E-01 |
|  |  |  |  | UNAFF | G | A | 30/139/128 | 0.47 | 0.45 | 4.36E-01 |
