# Supplementary material for: CYP1A1 Ile462Val polymorphism and colorectal cancer risk in Polish patients
Source: Med Oncol. 2014 Jun 18;31(7):72. doi: 10.1007/s12032-014-0072-y (PMC4079939; doi:10.1007/s12032-014-0072-y)
Supplement: Supplementary file 17 — Supplementary material 17 (DOCX 20 kb) [file 12032_2014_72_MOESM17_ESM.docx]

Supplementary Table 6. Hardy-Weinberg equilibrium for the combined Warsaw Center of Oncology – Institute (COI) and Wroclaw Medical University (WMU) cohort. Minor allele (A1); major allele (A2).

| **SNP** | **Chr.** | **Pos. NCBI Build 37** | **Gene** | **Test** | **A1** | **A2** | **GENOTYPES** | **O(HET)** | **E(HET)** | **p-value** |
| --- | --- | --- | --- | --- | --- | --- | --- | --- | --- | --- |
| rs2279017 | 3 | 14190237 | XPC | ALL | T | G | 143/415/315 | 0.48 | 0.48 | 7.78E-01 |
|  |  |  |  | AFF | T | G | 70/232/164 | 0.50 | 0.48 | 4.41E-01 |
|  |  |  |  | UNAFF | T | G | 73/179/147 | 0.45 | 0.48 | 1.77E-01 |
| rs1208 | 8 | 18258316 | NAT2 | ALL | G | A | 148/424/308 | 0.48 | 0.48 | 9.44E-01 |
|  |  |  |  | AFF | G | A | 76/247/151 | 0.52 | 0.49 | 1.57E-01 |
|  |  |  |  | UNAFF | G | A | 70/172/156 | 0.43 | 0.48 | 7.34E-02 |
| rs861539 | 14 | 104165753 | XRCC3 | ALL | A | G | 103/398/376 | 0.45 | 0.45 | 9.40E-01 |
|  |  |  |  | AFF | A | G | 55/222/192 | 0.47 | 0.46 | 4.81E-01 |
|  |  |  |  | UNAFF | A | G | 47/170/183 | 0.43 | 0.44 | 4.30E-01 |
| rs1048943 | 15 | 75012985 | CYP1A1 | ALL | C | T | 5/91/788 | 0.10 | 0.11 | 1.99E-01 |
|  |  |  |  | AFF | C | T | 3/59/414 | 0.12 | 0.13 | 4.72E-01 |
|  |  |  |  | UNAFF | C | T | 2/29/369 | 0.07 | 0.08 | 1.37E-01 |
| rs11615 | 19 | 45923653 | ERCC1 | ALL | G | A | 118/417/343 | 0.47 | 0.47 | 6.65E-01 |
|  |  |  |  | AFF | G | A | 71/226/176 | 0.48 | 0.48 | 1.00E+00 |
|  |  |  |  | UNAFF | G | A | 46/187/164 | 0.47 | 0.46 | 5.82E-01 |
