# Supplementary material for: CYP1A1 Ile462Val polymorphism and colorectal cancer risk in Polish patients
Source: Med Oncol. 2014 Jun 18;31(7):72. doi: 10.1007/s12032-014-0072-y (PMC4079939; doi:10.1007/s12032-014-0072-y)
Supplement: Supplementary file 18 — Supplementary material 18 (DOCX 22 kb) [file 12032_2014_72_MOESM18_ESM.docx]

Supplementary Table 7. Marker allele association for the Wroclaw Medical University (WMU) cohort. All (A); females (B); males (C). Minor allele (A1); major allele (A2).

A)

| **SNP** | **Chr.** | **Pos. NCBI (hg19)** | **Gene** | **A1** | **A1_Affected** | **A1_Unaffected** | **A2** | **OR (95% CI)** | **p-value (Fisher ex. test)** | **p-value _cor._ Bonf.** | **p-value _cor._ BH** |
| --- | --- | --- | --- | --- | --- | --- | --- | --- | --- | --- | --- |
| rs2279017 | 3 | 14190237 | XPC | T | 0.48 | 0.40 | G | 1.37 (0.93-2.03) | 1.14E-01 | 5.71E-01 | 1.90E-01 |
| rs1208 | 8 | 18258316 | NAT2 | G | 0.47 | 0.34 | A | 1.75 (1.18-2.6) | 5.43E-03 | 2.72E-02 | 2.72E-02 |
| rs861539 | 14 | 104165753 | XRCC3 | A | 0.33 | 0.31 | G | 1.06 (0.7-1.6) | 8.34E-01 | 1.00E+00 | 1.00E+00 |
| rs1048943 | 15 | 75012985 | CYP1A1 | C | 0.14 | 0.07 | T | 2.25 (1.14-4.44) | 2.32E-02 | 1.16E-01 | 5.79E-02 |
| rs11615 | 19 | 45923653 | ERCC1 | G | 0.40 | 0.40 | A | 0.99 (0.67-1.46) | 1.00E+00 | 1.00E+00 | 1.00E+00 |

B)

| **SNP** | **Chr.** | **Pos. NCBI (hg19)** | **Gene** | **A1** | **A1_Affected** | **A1_Unaffected** | **A2** | **OR (95% CI)** | **p-value (Fisher ex. test)** | **p-value _cor._ Bonf.** | **p-value _cor._ BH** |
| --- | --- | --- | --- | --- | --- | --- | --- | --- | --- | --- | --- |
| rs2279017 | 3 | 14190237 | XPC | T | 0.48 | 0.41 | G | 1.3 (0.77-2.2) | 3.50E-01 | 1.00E+00 | 4.66E-01 |
| rs1208 | 8 | 18258316 | NAT2 | G | 0.48 | 0.36 | A | 1.61 (0.95-2.72) | 8.20E-02 | 3.28E-01 | 1.64E-01 |
| rs861539 | 14 | 104165753 | XRCC3 | A | 0.29 | 0.33 | G | 0.82 (0.47-1.46) | 5.66E-01 | 1.00E+00 | 5.66E-01 |
| rs1048943 | 15 | 75012985 | CYP1A1 | C | 0.11 | 0.04 | T | 2.89 (1.03-8.11) | 4.27E-02 | 1.71E-01 | 1.64E-01 |
| rs11615 | 19 | 45923653 | ERCC1 | G | 0.36 | 0.37 | A | 0.97 (0.57-1.66) | 1.00E+00 | 1.00E+00 | 1.00E+00 |

C)

| **SNP** | **Chr.** | **Pos. NCBI (hg19)** | **Gene** | **A1** | **A1_Affected** | **A1_Unaffected** | **A2** | **OR (95% CI)** | **p-value (Fisher ex. test)** | **p-value _cor._ Bonf.** | **p-value _cor._ BH** |
| --- | --- | --- | --- | --- | --- | --- | --- | --- | --- | --- | --- |
| rs2279017 | 3 | 14190237 | XPC | T | 0.48 | 0.36 | G | 1.6 (0.84-3.04) | 1.98E-01 | 9.92E-01 | 4.96E-01 |
| rs1208 | 8 | 18258316 | NAT2 | G | 0.47 | 0.28 | A | 2.31 (1.17-4.53) | 1.55E-02 | 7.76E-02 | 7.76E-02 |
| rs861539 | 14 | 104165753 | XRCC3 | A | 0.35 | 0.28 | G | 1.44 (0.73-2.86) | 3.15E-01 | 1.00E+00 | 5.25E-01 |
| rs1048943 | 15 | 75012985 | CYP1A1 | C | 0.15 | 0.12 | T | 1.32 (0.52-3.34) | 6.54E-01 | 1.00E+00 | 6.54E-01 |
| rs11615 | 19 | 45923653 | ERCC1 | G | 0.42 | 0.47 | A | 0.83 (0.44-1.55) | 6.31E-01 | 1.00E+00 | 6.54E-01 |
