# Supplementary material for: CYP1A1 Ile462Val polymorphism and colorectal cancer risk in Polish patients
Source: Med Oncol. 2014 Jun 18;31(7):72. doi: 10.1007/s12032-014-0072-y (PMC4079939; doi:10.1007/s12032-014-0072-y)
Supplement: Supplementary file 19 — Supplementary material 19 (DOCX 23 kb) [file 12032_2014_72_MOESM19_ESM.docx]

Supplementary Table 8. Marker allele association for the Wroclaw Medical University (WMU) patients 50 years of age or above. All (A); females (B); males (C). Minor allele (A1); major allele (A2).

A)

| **SNP** | **Chr.** | **Pos. NCBI (hg19)** | **Gene** | **A1** | **A1_Affected** | **A1_Unaffected** | **A2** | **OR (95% CI)** | **p-value (Fisher ex. test)** | **p-value _cor._ Bonf.** | **p-value _cor._ BH** |
| --- | --- | --- | --- | --- | --- | --- | --- | --- | --- | --- | --- |
| rs2279017 | 3 | 14190237 | XPC | T | 0.48 | 0.40 | G | 1.42 (0.95-2.11) | 1.05E-01 | 5.27E-01 | 1.76E-01 |
| rs1208 | 8 | 18258316 | NAT2 | G | 0.49 | 0.34 | A | 1.84 (1.23-2.76) | 3.23E-03 | 1.62E-02 | 1.62E-02 |
| rs861539 | 14 | 104165753 | XRCC3 | A | 0.33 | 0.31 | G | 1.06 (0.69-1.61) | 8.30E-01 | 1.00E+00 | 1.00E+00 |
| rs1048943 | 15 | 75012985 | CYP1A1 | C | 0.13 | 0.07 | T | 2.2 (1.1-4.39) | 2.96E-02 | 1.48E-01 | 7.39E-02 |
| rs11615 | 19 | 45923653 | ERCC1 | G | 0.40 | 0.40 | A | 1.01 (0.68-1.5) | 1.00E+00 | 1.00E+00 | 1.00E+00 |

B)

| **SNP** | **Chr.** | **Pos. NCBI (hg19)** | **Gene** | **A1** | **A1_Affected** | **A1_Unaffected** | **A2** | **OR (95% CI)** | **p-value (Fisher ex. test)** | **p-value _cor._ Bonf.** | **p-value _cor._ BH** |
| --- | --- | --- | --- | --- | --- | --- | --- | --- | --- | --- | --- |
| rs2279017 | 3 | 14190237 | XPC | T | 0.49 | 0.41 | G | 1.35 (0.79-2.31) | 2.77E-01 | 1.00E+00 | 4.62E-01 |
| rs1208 | 8 | 18258316 | NAT2 | G | 0.48 | 0.36 | A | 1.6 (0.93-2.74) | 9.99E-02 | 4.99E-01 | 2.50E-01 |
| rs861539 | 14 | 104165753 | XRCC3 | A | 0.27 | 0.33 | G | 0.77 (0.43-1.38) | 4.61E-01 | 1.00E+00 | 5.76E-01 |
| rs1048943 | 15 | 75012985 | CYP1A1 | C | 0.12 | 0.04 | T | 3.11 (1.11-8.74) | 3.67E-02 | 1.84E-01 | 1.84E-01 |
| rs11615 | 19 | 45923653 | ERCC1 | G | 0.36 | 0.37 | A | 0.93 (0.54-1.62) | 8.89E-01 | 1.00E+00 | 8.89E-01 |

C)

| **SNP** | **Chr.** | **Pos. NCBI (hg19)** | **Gene** | **A1** | **A1_Affected** | **A1_Unaffected** | **A2** | **OR (95% CI)** | **p-value (Fisher ex. test)** | **p-value _cor._ Bonf.** | **p-value _cor._ BH** |
| --- | --- | --- | --- | --- | --- | --- | --- | --- | --- | --- | --- |
| rs2279017 | 3 | 14190237 | XPC | T | 0.48 | 0.36 | G | 1.64 (0.85-3.15) | 1.45E-01 | 7.27E-01 | 3.64E-01 |
| rs1208 | 8 | 18258316 | NAT2 | G | 0.49 | 0.28 | A | 2.53 (1.28-5.02) | 8.59E-03 | 4.30E-02 | 4.30E-02 |
| rs861539 | 14 | 104165753 | XRCC3 | A | 0.37 | 0.28 | G | 1.52 (0.76-3.03) | 3.04E-01 | 1.00E+00 | 5.07E-01 |
| rs1048943 | 15 | 75012985 | CYP1A1 | C | 0.14 | 0.12 | T | 1.21 (0.47-3.14) | 8.15E-01 | 1.00E+00 | 8.15E-01 |
| rs11615 | 19 | 45923653 | ERCC1 | G | 0.44 | 0.47 | A | 0.89 (0.47-1.69) | 7.47E-01 | 1.00E+00 | 8.15E-01 |
