# Supplementary material for: CYP1A1 Ile462Val polymorphism and colorectal cancer risk in Polish patients
Source: Med Oncol. 2014 Jun 18;31(7):72. doi: 10.1007/s12032-014-0072-y (PMC4079939; doi:10.1007/s12032-014-0072-y)
Supplement: Supplementary file 20 — Supplementary material 20 (DOCX 21 kb) [file 12032_2014_72_MOESM20_ESM.docx]

Supplementary Table 9. Marker allele association for the Warsaw Center of Oncology – Institute (COI) cohort. All (A); females (B); males (C). Minor allele (A1); major allele (A2).

A)

| **SNP** | **Chr.** | **Pos. NCBI (hg19)** | **Gene** | **A1** | **A1_Affected** | **A1_Unaffected** | **A2** | **OR (95% CI)** | **p-value (Fisher ex. test)** | **p-value _cor._ Bonf.** | **p-value _cor._ BH** |
| --- | --- | --- | --- | --- | --- | --- | --- | --- | --- | --- | --- |
| rs2279017 | 3 | 14190237 | XPC | T | 0.38 | 0.41 | G | 0.87 (0.69-1.08) | 2.12E-01 | 1.00E+00 | 3.56E-01 |
| rs1208 | 8 | 18258316 | NAT2 | G | 0.41 | 0.41 | A | 0.98 (0.79-1.23) | 9.11E-01 | 1.00E+00 | 9.11E-01 |
| rs861539 | 14 | 104165753 | XRCC3 | A | 0.36 | 0.34 | G | 1.12 (0.89-1.41) | 3.54E-01 | 1.00E+00 | 4.43E-01 |
| rs1048943 | 15 | 75012985 | CYP1A1 | C | 0.05 | 0.03 | T | 1.46 (0.83-2.55) | 2.14E-01 | 1.00E+00 | 3.56E-01 |
| rs11615 | 19 | 45923653 | ERCC1 | G | 0.39 | 0.34 | A | 1.25 (1-1.57) | 5.09E-02 | 2.54E-01 | 2.54E-01 |

B)

| **SNP** | **Chr.** | **Pos. NCBI (hg19)** | **Gene** | **A1** | **A1_Affected** | **A1_Unaffected** | **A2** | **OR (95% CI)** | **p-value (Fisher ex. test)** | **p-value _cor._ Bonf.** | **p-value _cor._ BH** |
| --- | --- | --- | --- | --- | --- | --- | --- | --- | --- | --- | --- |
| rs2279017 | 3 | 14190237 | XPC | T | 0.36 | 0.43 | G | 0.75 (0.57-0.98) | 3.99E-02 | 2.00E-01 | 2.00E-01 |
| rs1208 | 8 | 18258316 | NAT2 | G | 0.41 | 0.41 | A | 1 (0.77-1.31) | 1.00E+00 | 1.00E+00 | 1.00E+00 |
| rs861539 | 14 | 104165753 | XRCC3 | A | 0.35 | 0.35 | G | 1 (0.76-1.31) | 1.00E+00 | 1.00E+00 | 1.00E+00 |
| rs1048943 | 15 | 75012985 | CYP1A1 | C | 0.05 | 0.03 | T | 1.96 (0.95-4.05) | 8.31E-02 | 4.16E-01 | 2.08E-01 |
| rs11615 | 19 | 45923653 | ERCC1 | G | 0.39 | 0.36 | A | 1.14 (0.87-1.5) | 3.67E-01 | 1.00E+00 | 6.11E-01 |

C)

| **SNP** | **Chr.** | **Pos. NCBI (hg19)** | **Gene** | **A1** | **A1_Affected** | **A1_Unaffected** | **A2** | **OR (95% CI)** | **p-value (Fisher ex. test)** | **p-value _cor._ Bonf.** | **p-value _cor._ BH** |
| --- | --- | --- | --- | --- | --- | --- | --- | --- | --- | --- | --- |
| rs2279017 | 3 | 14190237 | XPC | T | 0.40 | 0.36 | G | 1.19 (0.8-1.78) | 4.17E-01 | 1.00E+00 | 6.96E-01 |
| rs1208 | 8 | 18258316 | NAT2 | G | 0.40 | 0.41 | A | 0.95 (0.64-1.4) | 8.41E-01 | 1.00E+00 | 8.41E-01 |
| rs861539 | 14 | 104165753 | XRCC3 | A | 0.38 | 0.30 | G | 1.46 (0.97-2.2) | 7.95E-02 | 3.97E-01 | 1.99E-01 |
| rs1048943 | 15 | 75012985 | CYP1A1 | C | 0.04 | 0.05 | T | 0.86 (0.35-2.13) | 8.18E-01 | 1.00E+00 | 8.41E-01 |
| rs11615 | 19 | 45923653 | ERCC1 | G | 0.39 | 0.29 | A | 1.58 (1.05-2.38) | 3.12E-02 | 1.56E-01 | 1.56E-01 |
